# Early and sustained efficacy of fremanezumab over 24-weeks in migraine patients with multiple preventive treatment failures: the multicenter, prospective, real-life FRIEND2 study

**DOI:** 10.1186/s10194-023-01561-w

**Published:** 2023-03-23

**Authors:** Piero Barbanti, Gabriella Egeo, Cinzia Aurilia, Paola Torelli, Cinzia Finocchi, Florindo d’Onofrio, Luigi d’Onofrio, Renata Rao, Stefano Messina, Laura Di Clemente, Angelo Ranieri, Massimo Autunno, Giuliano Sette, Bruno Colombo, Antonio Carnevale, Marco Aguggia, Miriam Tasillo, Francesco Zoroddu, Fabio Frediani, Massimo Filippi, Carlo Tomino, Stefania Proietti, Stefano Bonassi, for the FRIEND-Study Group, Maria Albanese, Marco Bertolini, Davide Bertuzzo, Maria Bloise, Francesco Bono, Laura Borrello, Cecilia Camarda, Giulia Fiorentini, Licia Grazzi, Domenica Le Pera, Roberta Messina, Pietro Querzani, Antonio Salerno, Silvia Strumia, Alessandro Valenza, Fabrizio Vernieri, Giovanna Viticchi

**Affiliations:** 1 Headache and Pain Unit, IRCCS San Raffaele Roma, Via Della Pisana 235, 00163 Rome, Italy; 2 San Raffaele University, Rome, Italy; 3 Department of Medicine and Surgery, Headache Center, Neurology Unit, University of Parma, Parma, Italy; 4 Neurology Unit, San Paolo Hospital, ASL 2, Savona, Italy; 5 Neurology Unit, San Giuseppe Moscati Hospital, Avellino, Italy; 6 Campus Bio-Medico University Hospital, Rome, Italy; 7 Department of Vision and Neurological Sciences, Spedali Civili, Brescia, Italy; 8 Department of Neurology-Stroke Unit, Laboratory of Neuroscience, Istituto Auxologico Italiano, IRCCS, Milano, Italy; 9 Headache Center, Neurology Unit, San Camillo-Forlanini Hospital, Rome, Italy; 10 Neurology Unit and Stroke-Unit, AORN A. Cardarelli, Naples, Italy; 11 Department of Clinical and Experimental Medicine, University of Messina, Messina, Italy; 12 Department of Neuroscience, Mental Health and Sensory Organs (NESMOS), “Sapienza” University of Rome, Sant’Andrea University Hospital, Rome, Italy; 13 Department of Neurology, Headache Unit, Scientific Institute San Raffaele Hospital, Vita-Salute University, Milan, Italy; 14 Headache Center, Neurology Unit, San Filippo Neri Hospital, Rome, Italy; 15 Neurology and Stroke Unit, Cardinal Massaia Hospital, Asti, Italy; 16 Stroke Unit, S. Camillo de Lellis Hospital, Rieti, Italy; 17 Pediatric Headache Center, Neurology Unit, University of Sassari, Sassari, Italy; 18 Headache Center, ASST Santi Paolo Carlo, Milan, Italy; 19 Clinical and Molecular Epidemiology, IRCCS San Raffaele Roma, Rome, Italy; 20 Department of Human Sciences and Quality of Life Promotion, San Raffaele University, Rome, Italy

**Keywords:** Fremanezumab, Migraine treatment, CGRP monoclonal antibody, Real-world, Long-term treatment

## Abstract

**Background:**

To verify the long-term (24-week) efficacy, safety, and tolerability of fremanezumab in real-life patients with high-frequency episodic migraine (HFEM: ≥ 8 days/month) or chronic migraine (CM: ≥ 15 days/month), and multiple preventive treatment failures.

**Methods:**

This is a prospective, cohort, real-life study at 28 headache centers on consecutive patients affected by HFEM or CM with multiple preventive treatment failures who were prescribed subcutaneous fremanezumab (225 mg monthly/675 mg quarterly) for ≥ 24 weeks. Primary endpoint was the change in monthly migraine days (MMDs) in HFEM and monthly headache days (MHDs) in CM at weeks 21–24 compared to baseline. Secondary endpoints encompassed changes in monthly analgesic medications, ≥ 50%, ≥ 75%, and 100% responder rates, and variation in NRS, HIT-6 and MIDAS scores at the same time interval. Changes in MMDs/MHDs, monthly analgesic medications, ≥ 50%, ≥ 75%, and 100% responder rates, and variation in NRS and HIT-6 scores at week 4 were also monitored.

**Results:**

Four hundred ten patients who had received ≥ 1 dose of fremanezumab were considered for safety analysis while 148 patients treated for ≥ 24 weeks were included in the efficacy analysis. At weeks 21–24, fremanezumab significantly (*p* < 0.001) reduced MMDs, MHDs, monthly analgesic medications and NRS, HIT-6, and MIDAS scores in both HFEM and CM compared to baseline. The proportions of ≥ 50%, ≥ 75% and 100% responders at weeks 21-24were 75.0%, 30.8%, 9.6% (HFEM), and 72.9, 44.8 and 1% (CM). A significant (*p* < 0.001) decrease in MMDs, MHDs, monthly analgesic medications and NRS, HIT-6, and MIDAS scores in both HFEM and CM was already present at week 4. The proportions of ≥ 50%, ≥ 75%, and 100% responders at week 4 were 67.6%, 32.4%, 11.8% (HFEM) and 67.3%, 40%, 1.8% (CM). CM remitted to episodic migraine and medication overuse to no-medication overuse in 83.3 and 75% of patients at week 24, and in 80 and 72.4% at week 4. Adverse events were rare (2.4%), mild and transient. No patient discontinued treatment for any reason.

**Conclusions:**

Fremanezumab is characterized by an early and sustained efficacy in HFEM and CM patients with multiple preventive treatment failures in real-life, revealing an optimal safety and tolerability profile.

**Supplementary Information:**

The online version contains supplementary material available at 10.1186/s10194-023-01561-w.

## Introduction

Monoclonal antibodies (mAbs) targeting the calcitonin gene-related peptide (CGRP) pathway are among the most useful therapeutic tools for migraine prevention due to a favorable efficacy/tolerability profile coupled to a considerable speed of action [1]. Prospective, multicenter, real-world evidence (RWE) studies hinted that their effectiveness is higher than efficacy, suggesting that unilateral pain (alone or associated with unilateral cranial autonomic symptoms or allodynia) may represent a positive response predictor [2–5].

Fremanezumab is a humanized anti-CGRP mAb characterized by a flexible dosing regimen (monthly, quarterly) which proved effective in randomized, placebo-controlled trials (RCTs) in episodic migraine (HALO-EM study) and chronic migraine (HALO-CM study), with or without medication overuse, also in patients with prior therapeutic failures (FOCUS study) [6–8].

RCTs finding were confirmed also under real-world conditions in more complex and multifaceted patients, better representing the everyday clinical practice. In the 12-week, RWE, FRIEND study (FRemanezumab In rEal world evideNce stuDy) we documented a very good short-term efficacy and tolerability of fremanezumab in 53 patients affected by high-frequency episodic migraine (HFEM: ≥ 8 days/month) or chronic migraine (CM: ≥ 15 days/month) [9, 10].

In the present FRIEND2 study, we report the long-term (24-week) efficacy, safety, and tolerability of fremanezumab in a real-life, multicenter, prospective, cohort study on a larger population (*n* = 148) of patients affected by HFEM or CM with multiple therapeutic failures and diverse comorbidities.

## Methods

This is a multicenter, prospective, cohort, real-life study carried out at 28 headache centers across 10 Italian regions (Lombardy, Piedmont, Liguria, Emilia-Romagna, Marche, Latium, Sardinia, Campania, Calabria, and Sicily). The study is a part of the I-NEED project (Italian New migrainE Drugs database) and represents a sub-study of the large Italian Migraine Registry (I-GRAINE). We considered all consecutive, anti-CGRP mAbs naïve patients affected by HFEM or CM consecutively seen from July 28^th^ 2020who were prescribed subcutaneous fremanezumab 225 mg monthly or 675 mg quarterly– according to their preference—for at ≥ 24 weeks. All subjects had previously failed at least 3 preventive medications classes between tryciclics, anticonvulsants, and beta-blockers (or onabotulinum toxin A for those with CM), according to requirements of the Italian medicines agency (AIFA, Agenzia Italiana del Farmaco) [11].

We excluded patients with use of onabotulinum toxin A during the previous 12 weeks, prior exposure to anti-CGRP mAbs or with clinically significant cardiovascular disorders. No additional prophylactic medications were added during the study.

Specifically trained, board-certified neurologists gathered detailed information on sociodemographic and clinical characteristics of the patients via face-to-face interviews using a shared semi-structured questionnaire [9]. Each patient was asked to monitor migraine/headache days, pain severity (using the Numerical Rating Scale, NRS), monthly analgesic medications and migraine disability (using the Headache Impact Test, HIT-6, and the Migraine Disability Assessment Scale, MIDAS) during a 28-day run-in period and throughout the study, using a paper–pencil diary. Patient were also invited to report the occurrence of any adverse event.

All participating headache centers collected patient’s clinical data at weeks 12 and 24, in compliance with AIFA regulations [11]. Some centers (*n* = 7) monitored fremanezumab treatment effects on a monthly basis, according to their routine practice.

The primary endpoint was the change in the number of monthly migraine days (MMDs) for HFEM and of monthly headache days (MHDs) for CM at weeks 21–24 compared to baseline. In people with CM, the expression “headache day” refers to any headache day, encompassing both migraine-like or tension-type like headache.

Secondary endpoints included changes in monthly analgesic medications, ≥ 50%, ≥ 75%, and 100% responder rates and variation in NRS, HIT-6 and MIDAS scores at weeks 21–24 compared to baseline. Changes in MMDs/MHDs, monthly analgesic medications, ≥ 50%, ≥ 75%, and 100% responder rates, and variation in NRS and HIT-6 scores at week 4were also assessed.

All patients provided written informed consent before their study participation. The study received the approval from the IRCCS San Raffaele Roma Institutional Review Board (RP 19/26) and was mutually recognized by the other local Institutional Review Boards. The FRIEND2 study was not preregistered on any study registry site.

### Statistical methods

Descriptive statistics were reported as frequency and percentage for categorical variables, and as mean and standard deviation (SD) for continuous variables. Kolmogorov–Smirnov/Shapiro–Wilk test were applied to check departure from normality for quantitative variables. The chi-square test was used to compare frequencies between categorial variables, while Fisher's exact test was adopted when the expected frequency was < 5. The *t*-test for independent samples or the non-parametric Mann–Whitney U test were applied for inter-group comparisons (HFEM, CM), while *t*-test for paired samples or Wilcoxon's test were used to compare study endpoints before and after the treatment. Due to the exploratory nature of the study, no correction was applied for multiple comparisons; p-value < 0.05 was considered a statistically significant result. Sensitivity analyses were carried out excluding one clinical center at a time and examining the impact of the removal on the summary treatment effect. Statistical analyses were performed using the SPSS package program version 28.0.

## Results

**Fig. 1:**
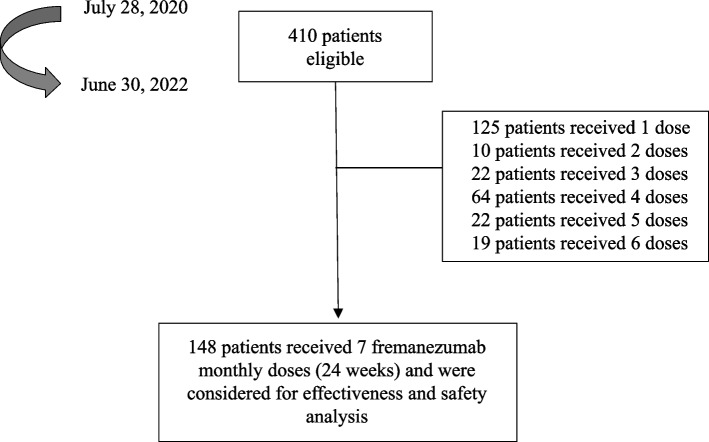
Patients’ disposition

As of June 30, 2022, 410 patients had received at least 1 dose of fremanezumab and were considered for safety analysis (HFEM/CM: 214/196; F/M: 340/70; age 48.9 ± 11.6 years) (Fig. 1). The efficacy analysis was performed on the 148 patients treated with fremanezumab for at least 24 weeks (fremanezumab 225 mg monthly: 98 patients; fremanezumab625 mg quarterly:60 patients). Patients’ characteristics are described in the Table 1.

**Table 1:** Demographic and clinical features of patients with high-frequency episodic migraine (HFEM) or chronic migraine (CM)

	Number (%) or mean ± SD			
	**All patients**	**HFEM**	**CM**	***p*-value**
**Patients**	148	52 (35.1)	96 (64.9)	-
**Age,** *yrs*	47.6 ± 10.6	50.2 ± 9.8	46.2 ± 10.9	**0.030**
**Females**	117 (79.1)	37 (71.1)	80 (83.3)	0.127
**BMI**	23.6 ± 3.5	23.4 ± 2.8	23.7 ± 3.9	0.737
**Age at CM onset,** *yrs*	16.8 ± 7.0	16.3 ± 7.3	17.0 ± 6.9	0.589
**MMDs/MHDs at baseline**	18.8 ± 6.8	11.5 ± 1.7	22.7 ± 5.1	**< 0.001**
**NRS score**	8.1 ± 1.1	7.9 ± 1.0	8.2 ± 1.1	0.153
**Pain quality**				
*Pulsating*	72 (53.0)	23 (46.9)	49 (56.4)	0.559
*Pressing/tightening*	38 (27.9)	15 (30.6)	23 (26.4)	
*Other*	26 (19.1)	11 (22.5)	15 (17.2)	
**Unilateral pain**	86 (58.1)	31 (59.6)	55 (57.3)	0.663
**Unilateral cranial autonomic symptoms**	61 (44.9)	22 (44.9)	39 (44.8)	0.994
**Allodynia**	82 (55.4)	31 (59.6)	51 (53.1)	0.558
**Dopaminergic symptoms**	104 (93.7)	36 (92.3)	68 (94.4)	0.974
**Monthly analgesic medications**	18.9 ± 12.7	12.0 ± 3.3	22.5 ± 14.3	**< 0.001**
**MO**	69 (71.9)	-	69 (71.9)	-
**Duration of MO**, *yrs*	7.3 ± 14.8	-	7.3 ± 14.8	
**Triptan responders**	91 (61.5)	37 (71.1)	54 (56.3)	0.109
**Pts using concomitant prophylaxis**	46 (32.2)			
*Tricyclics*	19 (41.3)	5 (33.3)	14 (45.2)	0.877
*Anticonvulsants*	20 (43.5)	6 (40.0)	14 (45.2)	
*Calcium-channels antagonists*	1 (2.2)	-	1 (3.2)	
*Serotoninergic antagonists*	12 (26.1)	6 (40.0)	6 (19.4)	
*Onabotulinum toxin A*	2 (4.3)	1 (6.7)	1 (3.2)	
**Prior treatment failures**	4.3 ± 1.4	4.0 ± 1.2	4.4 ± 1.5	0.099
*3–4*	99 (66.9)	40 (76.9)	59 (61.5)	0.084
> *4*	49 (33.1)	12 (23.1)	37 (38.5)	
**Onabotulinum toxin A responders**^**a**^	9 (34.6)	5 (62.5)	4 (22.2)	0.122
**Pts with ≥ 1 comorbidity**	94 (63.5)	32 (61.5)	62 (64.6)	0.850
**Pts with psychiatric comorbidities**	51 (34.4)	17 (32.7)	34 (35.4)	0.879
**HIT-6 score**	67.3 ± 5.8	65.6 ± 6.8	68.3 ± 5.0	**0.008**
**MIDAS score**	80.3 ± 59.9	62.4 ± 42.0	90.0 ± 65.8	**0.023**
**Fremanezumab dosing regimen**				
*Monthly*	98 (66.2)	38 (73.1)	60 (62.5)	**-**
*Quarterly*	50 (33.8)	14 (26.9)	36 (37.5)	

*HFEM* High frequency episodic migraine, *CM* Chronic migraine, *BMI* Body mass index, *MMDs* Monthly migraine days, *MHDs* Monthly headache days, *NRS* Numerical rating scale, *MO* Medication overuse, *HIT-6* Headache Impact Test-6, *MIDAS* Migraine disability assessment scale

^a^Proportion calculated on the 26 subjects who were treated with onabotulinum toxin A

Subjects with CM were younger (*p* = 0.030), used more analgesics (*p* < 0.001), and had higher HIT-6 (*p* = 0.008) and MIDAS scores (*p* < 0.023) than those with HFEM. Patients’data at weeks 12 and 24 were available for all 148 patients, while additional findings at weeks 4, 8, 16, and 20 were available in a subgroup of 89 patients (60.1%). This patients’ subgroup had higher NRS (8.3 ± 1.0 *vs* 7.7 ± 1.1; *p* < 0.001) and HIT-6 scores (66.0 ± 7.2*vs* 68.2 ± 4.6; *p* = 0.028), and more therapeutic failures (4.6 ± 1.4*vs* 3.8 ± 1.3; *p* < 0.001).

### Primary efficacy endpoints

**Fig. 2:**
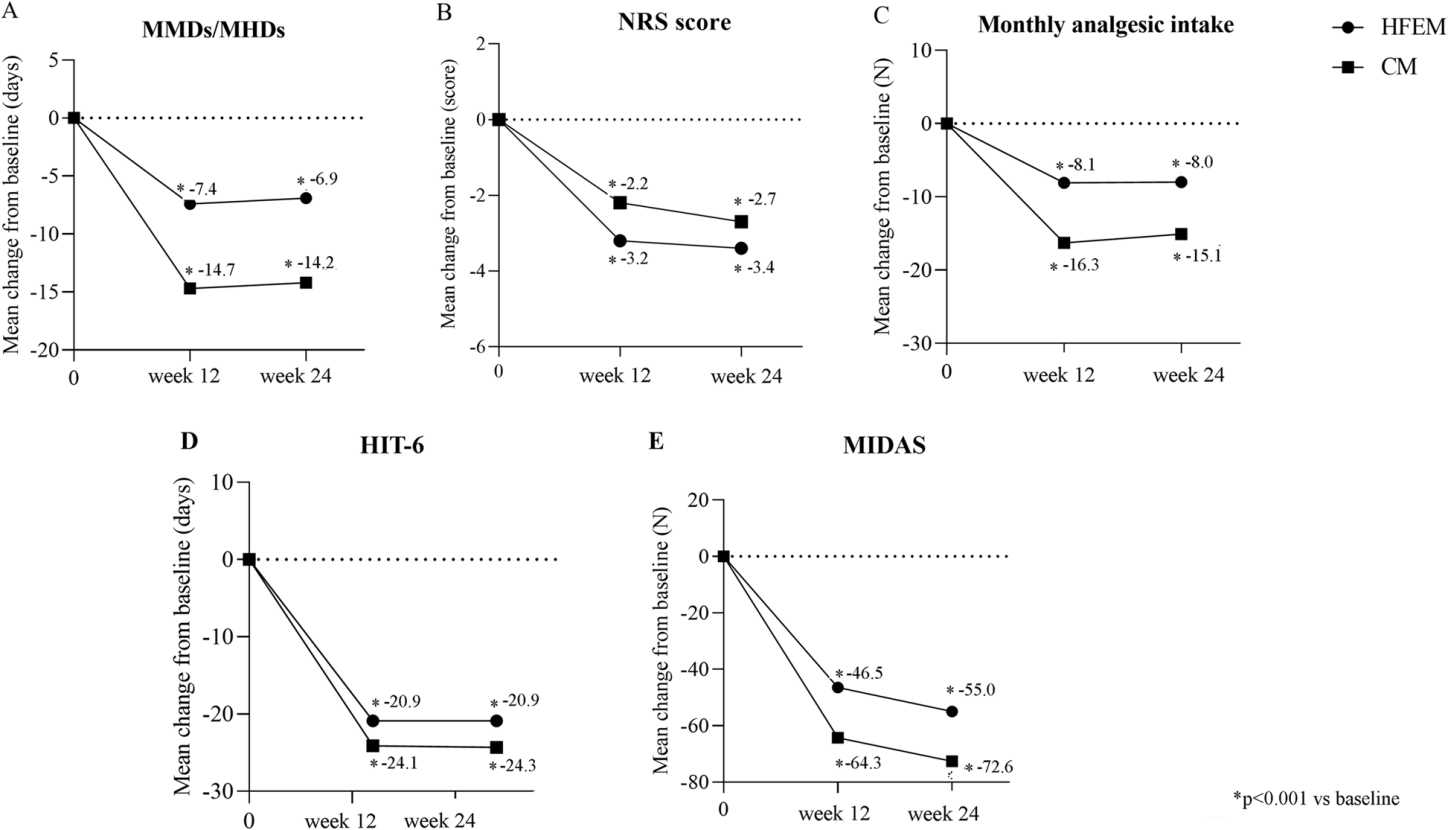
Mean change in (**A**) monthly migraine days/monthly headache days (MMDs/MHDs), **B** Numerical Rating Scale (NRS) score, **C** monthly analgesic medications, **D** Headache Impact Test-6 (HIT-6) score and (**E**) Migraine Disability Assessment Scale (MIDAS) score from baseline to week 24 in patients from headache centers providing efficacy data at week 12 and 24 (*n* = 148). HFEM, high-frequency episodic migraine; CM, chronic migraine

Fremanezumab was effective in reducing MMDs in HFEM (-6.9 ± 3.6, *p* < 0.001) and MHDs in CM (-14.2 ± 7.6, *p* < 0.001) at weeks 21–24 compared to baseline (Fig. 2; Supplementary table).

### Secondary efficacy endpoints

**Fig. 3:**
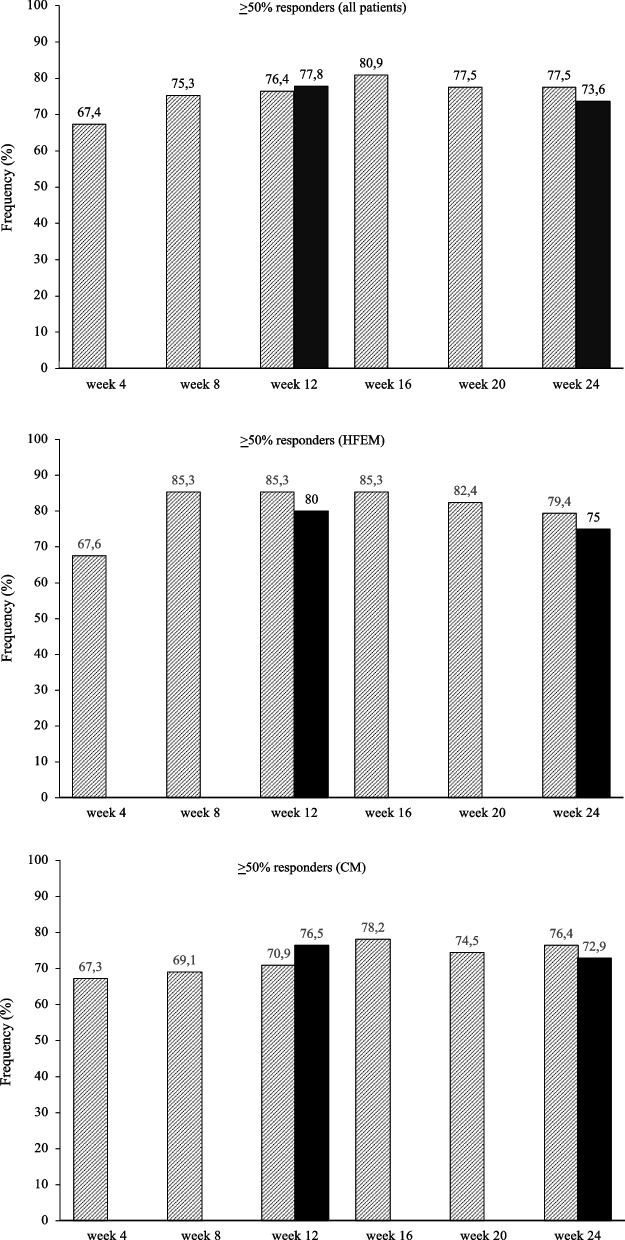
Proportion of patients with a 50% or greater reduction in monthly migraine/headache days. Black bars: patients from headache centersproviding data at weeks 12 and 24 (*n* = 148). Hatched bars: patients from headache centers providing data at weeks 4, 8, 12, 16, 20 and 24 (*n* = 89). HFEM: high-frequency episodic migraine; CM: chronic migraine

**Fig. 4:**
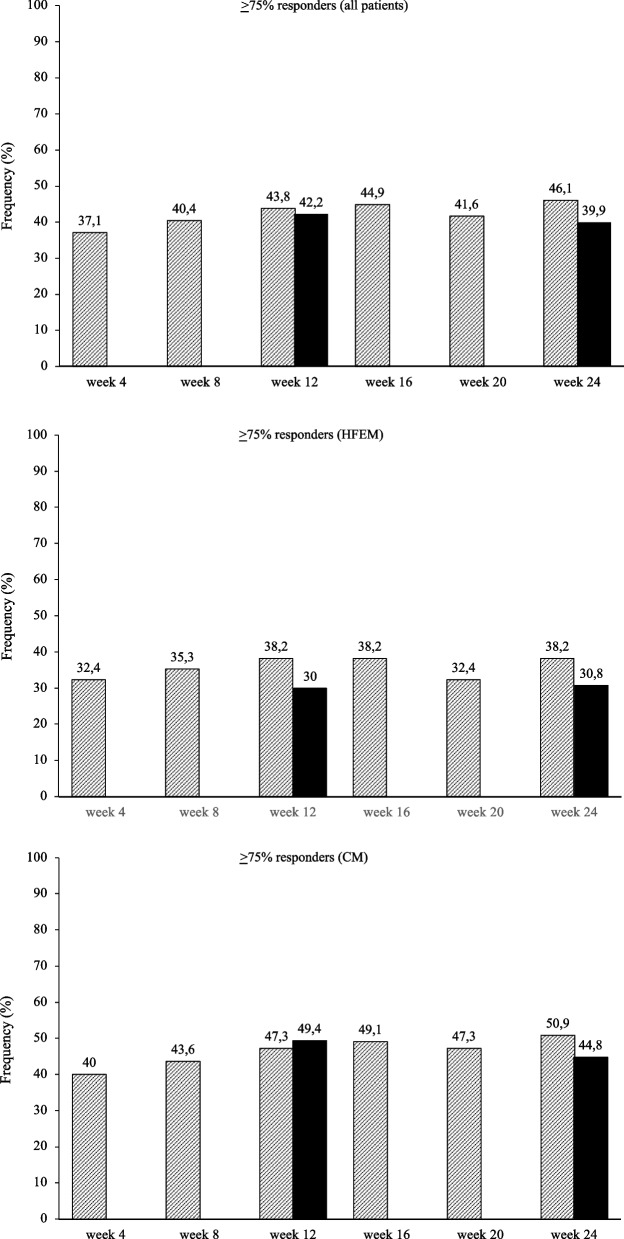
Proportion of patients with a 75% or greater reduction in monthly migraine/headache days. Black bars: patients from headache centers providing data at weeks 12 and 24 (*n* = 148). Hatched bars: patients from headache centers providing data at weeks 4, 8, 12, 16, 20 and 24 (*n* = 89). HFEM: high-frequency episodic migraine; CM: chronic migraine

**Fig. 5:**
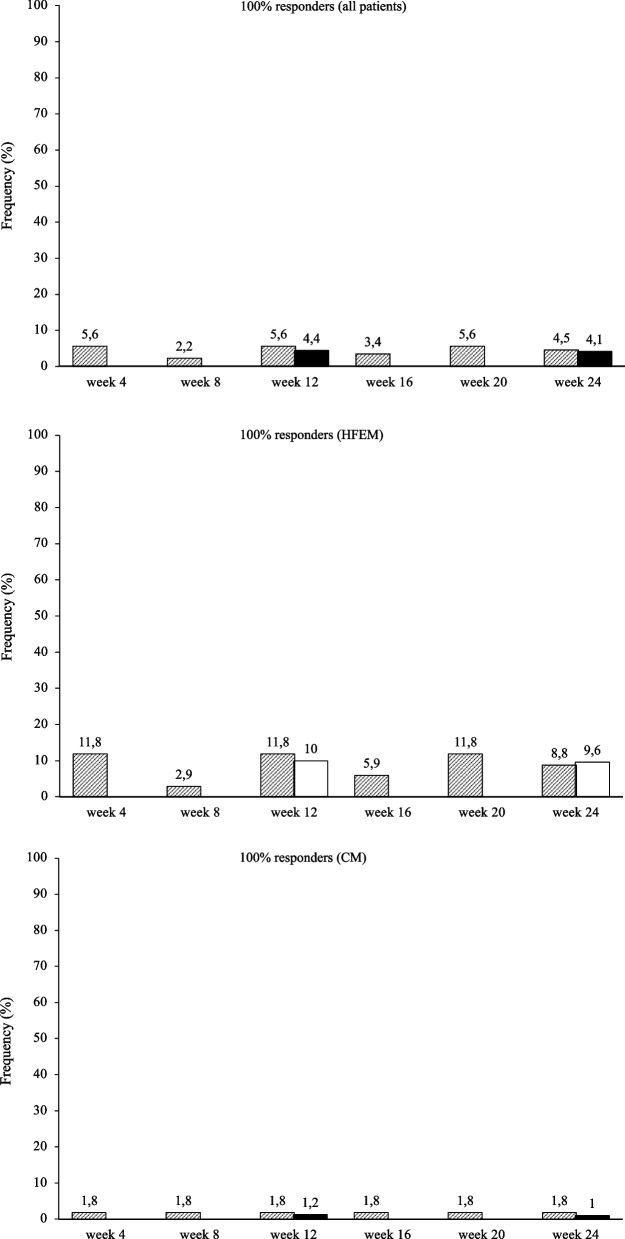
Proportion of patients with a 100% reduction in monthly migraine/headache days. Black bars: patients from headache centers providing data at weeks 12 and 24 (*n* = 148). Hatched bars: patients from headache centers providing data at weeks 4, 8, 12, 16, 20 and 24 (*n* = 89). HFEM: high-frequency episodic migraine; CM: chronic migraine

At the same time interval, fremanezumab proved effective (*p* < 0.001) in reducing NRS score (-3.4 ± 2.3; -2.7 ± 2.3), monthly analgesic medications (-8.0 ± 3.5; -15.1 ± 10.9), HIT-6 score (-20.9 ± 18.9; -24.3 ± 23.9), and MIDAS score (-55.0 ± 42.5; -72.6 ± 59.5) in both HFEM and CM (Fig. 2; Supplementary table). The proportion of ≥ 50%, ≥ 75% and 100% responders in HFEM was 75.0, 30.8 and 9.6%, while in CM was 72.9, 44.8 and 1% (Figs. 3, 4 and 5).

**Fig. 6:**
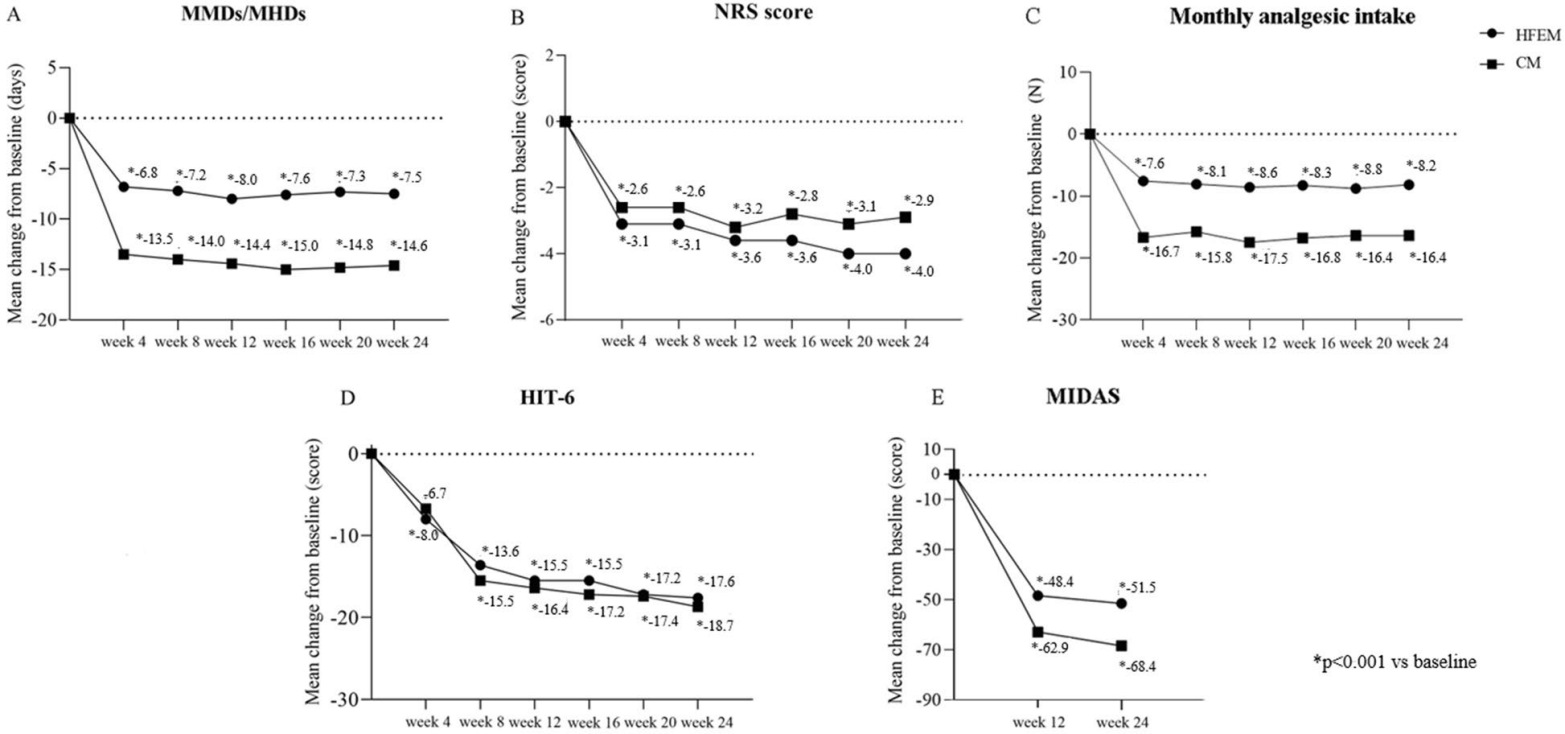
Mean change in (**A**) monthly migraine days/monthly headache days (MMDs/MHDs), **B** Numerical Rating Scale (NRS) score, **C** monthly analgesic medications, **D** Headache Impact Test-6 (HIT-6) score and (E) Migraine Disability Assessment Scale (MIDAS) score from baseline to week 24 in patients from headache centers providing efficacy data at weeks 4, 8, 12,16, 20 and 24 (*n* = 89). HFEM, high-frequency episodic migraine; CM, chronic migraine

At week 4, fremanezumab significantly (*p* < 0.001) improved MMDs/MHDs (-6.8 ± 3.2/-13.5 ± 8.5), monthly analgesic medications (-7.6 ± 4.1; -16.7 ± 12.8), NRS (-2.6 ± 2.3.; -3.1 ± 2.6) and HIT-6 scores (-6.7 ± 33.8; -8.0 ± 29.9) in patients with HFEM and CM (Fig. 6).The proportion of ≥ 50%, ≥ 75%, and 100% responders was 67.6, 32.4 and 11.8% in HFEM and 67.3, 40 and 1.8% in CM. These clinical benefits were sustained across the whole treatment period (Figs. 3, 4 and 5).

**Fig. 7:**
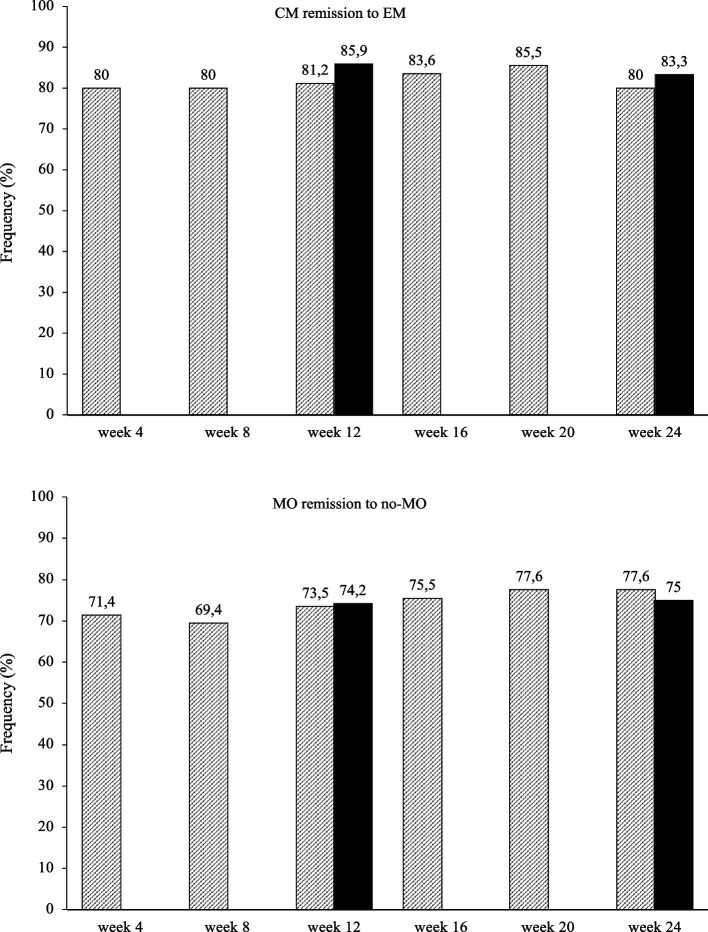
Proportion of patients remitting from chronic migraine (CM) to episodic migraine (EM) and from medication overuse (MO) to no medication overuse (no-MO). Black bars: patients from headache centers providing data at weeks 12 and 24 (*n* = 148). Hatched bars: patients from headache centers providing data at weeks 4, 8, 12, 16, 20 and 24 (*n* = 89)

Remission from CM to episodic migraine and from medication overuse to no-medication overuse occurred in 83.3 and 75% of the patients at week 24 (80 and 71.4%, respectively, at week 4) (Fig. 7).

Ten patients (2.4%) reported adverse events, rated as mild and transient: constipation (*n* = 1), injection site itch (*n* = 5), injection site edema (*n* = 4). No patient discontinued the treatment for any reason.

## Discussion

This 24-week, multicenter, real-life study carried out in patients with multiple preventive failures and diverse comorbidities, extends the findings of the 12-week FRIEND trial demonstrating that fremanezumab induces an early and sustained improvement in migraine frequency, pain severity, analgesic use, and disability, being characterized by a high proportion of responders (~ 75%) and super-responders (~ 40%) [9].

In the present RWE study, fremanezumab’s effectiveness is considerably greater than the efficacy reported in the 6 months open-label extension of the FOCUS trial, performed in adults with episodic or chronic migraine with documented prior inadequate response to 2 to 4 migraine preventative medication classes [12]. We found a significantly higher reduction in MMDs/MHDs (-6.9/-14.2 vs -4.8/-5.2), HIT-6 (-20.9/-24.3 vs -8.2/-8.0) and MIDAS (-55.0/-72.6 vs -27.9/-32.0) scores, a greater proportion of ≥ 50% responders (73.6%% vs 45.0%/46.0%) and ≥ 75% responders (39.9% vs 15.0%/20.0%) and a lower frequency of adverse events (2.5% vs 17.0%/20.0%) (Supplementary table; Fig. 2). These results are even more striking when considering that our patients had a more complex clinical scenario, showing at baseline higher monthly migraine/headache frequency (18.8 vs 14.2 days) and disability (MIDAS score: 80.3 vs 62.0) and a greater proportion of subjects who had failed at least 3 preventive treatments (93.3% vs 50.0%).

Different real-life studies highlighted that the effectiveness of anti-CGRP mAbs is higher than efficacy. The comparison of ≥ 50% responders at week 12 for episodic and chronic migraine in RWE vs RCTs is 59.4% vs 30.0% and 55.5% vs 41.0% for erenumab, 66.7% vs 41.8% and 66.7% vs 32.8% for galcanezumab, and 76.5% vs 43.0% and 58.3% vs 29.0% for monthly fremanezumab [3, 4, 13–17]. The reason is unclear and is still matter of speculation. Patients in real-life are more challenging and multifaceted than in RCTs, being characterized by higher migraine frequency, more frequent depressive comorbidities, and multiple therapeutic failures. These conditions are associated with an increased CGRP activity which could emphasize the therapeutic properties of anti-CGRP mAbs. In fact, interictal CGRP levels in peripheral blood progressively increase from episodic migraine to CM, while elevated CGRP-like immunoreactivity in the cerebrospinal fluid had been suggested as a trait marker of major depressive disorder [18, 19]. We also found a very low number of adverse events, in agreement with other real-life studies [3, 4, 13–17]. Some additional issues should be considered. Firstly, in a real-world setting spontaneously reported adverse events are usually recorded, whereas in RCTs all adverse events are carefully and extensively investigated by specific questioning. Secondly, in RWE, patients are likely to underestimate mild or moderate adverse events due to their prior exposure to different preventive treatments associated with multiple side effects.

Anti-CGRP mAbs demonstrate a remarkable speed of action—probably related to their kinetics and symptomatic effect –a quality which represents an advantage over conventional treatments [20]. A rapid efficacy onset has been reported for all mAbs targeting the CGRP pathway, particularly intravenous eptinezumab, effective also in shortening time to headache and most bothersome symptom freedom when administered during a migraine attack [6–8, 21–30].A clinically meaningful feature of the present study is the substantial equivalence of migraine improvement following fremanezumab administration at week 4 and at week 24 in terms of migraine frequency, analgesic use, pain severity, responder and super-responder rates, and remission from CM to episodic migraine and from medication overuse to no medication overuse. Fremanezumab reaches the maximum plasma concentration 5–7 days after a single administration, an effect not impacted by ethnicity and dose regimens [31]. This suggests that fremanezumab rapidly counteracts CGRP released by sensitized trigeminal endings, ultimately also exerting a symptomatic effect, at least at the beginning of the treatment. Over time, fremanezumab-induced desensitization progress centrally, accounting for a sustained preventive effect.

This study has some limitations. The global efficacy and tolerability of fremanezumab was evaluated without considering the dosing regimen. This choice was justified by the heterogeneous distribution of patients treated monthly or quarterly, especially in subjects affected by HFEM (73.1% vs 26.9%). Some headache centers—due to their internal rules—inquired treatment outcomes only at weeks 12 and 24, the timepoints established by AIFA [11] – rather than monthly. No substantial difference was observed with results from the centers reporting data monthly. We included patients with at least 8 MMDs, according to AIFA regulation, thus our results cannot be generalized to patients with lower migraine frequency. Further, we did not distinguish headache days from migraine days in CM patients, because this differentiation in a real-life CM population and setting may be challenging. Lastly, patients did not use electronic diaries, potentially reducing data reliability. Strengths of the present work are the size of patients’ sample, the large number of headache centers involved covering the 50% of the Italian regions, and its prospective design.

In conclusion, fremanezumab is rapidly effective in highly disabled migraine patients affected by HFEM or CM with multiple prior therapeutic failures, medication overuse and frequent comorbidities. The clinical benefit is appraisable during the first treatment month and is sustained over time. Adverse events are mild and rare.

## Supplementary Information

**Additional file 1: Supplementary table.** Change in monthly migraine days (MMDs), monthly headache days (MHDs), monthly analgesic medications (MAM), Numerical Rating Scale (NRS) score, Headache Impact Test-6 (HIT-6) score, and Migraine Disability Assessment Scale (MIDAS) score from baseline to weeks 9-12 and 21-24.

## Data Availability

Anonymized data will be shared by request from any qualified investigator.

## Abbreviations

CM: Chronic migraine
RCTs: Placebo-controlled trials
HFEM: High-frequency episodic
MMDs: Monthly migraine days
AIFA: Agenzia Italiana del Farmaco
NRS: Numerical Rating Scale
HIT-6: Headache Impact Test
MIDAS: Migraine Disability Assessment Scale
mAbs: Monoclonal antibodies
CGRP: Calcitonin gene-related peptide
EM: Episodic migraine
AEs: Adverse events
SD: Standard deviation
