# Supplementary material for: Early and sustained efficacy of fremanezumab over 24-weeks in migraine patients with multiple preventive treatment failures: the multicenter, prospective, real-life FRIEND2 study
Source: J Headache Pain. 2023 Mar 23;24(1):30. doi: 10.1186/s10194-023-01561-w (PMC10035286; doi:10.1186/s10194-023-01561-w)
Supplement: Supplementary file 1 — Additional file 1: Supplementary table. Change in monthly migraine days (MMDs), monthly headache days (MHDs), monthly analgesic medications (MAM), Numerical Rating Scale (NRS) score, Headache Impact Test-6 (HIT-6) score, and Migraine Disability Assessment Scale (MIDAS) score from baseline to weeks 9-12 and 21-24. [file 10194_2023_1561_MOESM1_ESM.docx]

**Supplementary table:** Change in monthly migraine days (MMDs), monthly headache days (MHDs), monthly analgesic medications (MAM), Numerical Rating Scale (NRS) score, Headache Impact Test-6 (HIT-6) score, and Migraine Disability Assessment Scale (MIDAS) score from baseline to weeks 9-12 and 21-24.

| **Variable** | **HFEM** | **CM** |
| --- | --- | --- |
| **MMDs/MHDs**  *Baseline*  *weeks 9-12*  *weeks 21-24*  *Mean change*  *weeks 9-12*  *weeks 21-24* | 11.6±1.7  4.2±2.9  4.6±3.6  -7.4±2.9, p<0.001  -6.9±3.6, p<0.001 | 22.7±5.2  8.0±6.7  8.6±6.7  -14.7±7.6, p<0.001  -14.2±7.6, p<0.001 |
| **NRS**  *Baseline*  *weeks 9-12*  *weeks 21-24*  *Mean change*  *weeks 9-12*  *weeks 21-24* | 7.9±1.0  4.8±2.2  4.5±2.0  -3.2±2.4, p<0.001  -3.4±2.3, p<0.001 | 8.1±1.2  5.9±5.8  5.5±2.2  -2.2±5.6, p<0.001  -2.7±2.3, p<0.001 |
| **MAM**  *Baseline*  *weeks 9-12*  *weeks 21-24*  *Mean change*  *weeks 9-12*  *weeks 21-24* | 12.0±3.4  3.9±2.9  4.1±2.6  -8.1±3.9, p<0.001  -8.0±3.5, p<0.001 | 23.0±14.9  6.6±6.0  7.4±8.9  -16.3±12.4, p<0.001  -15.1±10.9, p<0.001 |
| **HIT-6**  *Baseline*  *weeks 9-12*  *weeks 21-24*  *Mean change*  *weeks 9-12*  *weeks 21-24* | 66.5±4.1  45.5±20.5  44.8±19.0  -20.9±20.4, p<0.001  -20.9±18.9, p<0.001 | 68.6±4.4  44.6±22.6  43.9±23.0  -24.1±22.7, p<0.001  -24.3±23.9, p<0.001 |
| **MIDAS**  *Baseline*  *weeks 9-12*  *weeks 21-24*  *Mean change*  *weeks 9-12*  *weeks 21-24* | 62.4±42.0  13.3±15.6  7.7±9.7  **-**46.5±38.2, p<0.001  -55.0±42.5, p<0.001 | 90.0±65.8  20.6±45.9  17.4±29.5  **-**64.3±60.7, p<0.001  -72.6±59.5, p<0.001 |
